# Supplementary material for: Functional Synaptic Interactions and Inhibitory Circuitry of the PreBötzinger Complex in the Rhythmic Slice
Source: bioRxiv. 2026 May 24:2026.05.23.727419. Preprint. [Version 1] doi: 10.64898/2026.05.23.727419 (PMC13228582; doi:10.64898/2026.05.23.727419)
Supplement: 1 [file NIHPP2026.05.23.727419V1-supplement-1.pdf]

bioRxiv preprint doi: https://doi.org/10.64898/2026.05.23.727419; this version posted May 24, 2026. The copyright holder for this preprint (which was not certified by peer review) is the author/funder, who has granted bioRxiv a license to display the preprint in perpetuity. It is made available under a CC-BY 4.0 International license.

Supplemental Materials

Sensitivity Analysis of Reversal Potentials

The robustness of the synaptic conductance inference was evaluated by systematically varying the assumed reversal potentials for excitation (E_e) and inhibition (E_i) within biologically plausible ranges (Supplemental Figure 1). The resulting 3×3 matrix illustrates the reconstructed excitatory (G_{exc}, red) and inhibitory (G_{inh}, blue) conductances across combinations of E_e at -20.0, -10.0, and 0.0 mV, and E_i at -90.0, -80.0, and -70.0 mV. Each panel represents the mean conductance over two normalized respiratory cycles for a representative inspiratory neuron.

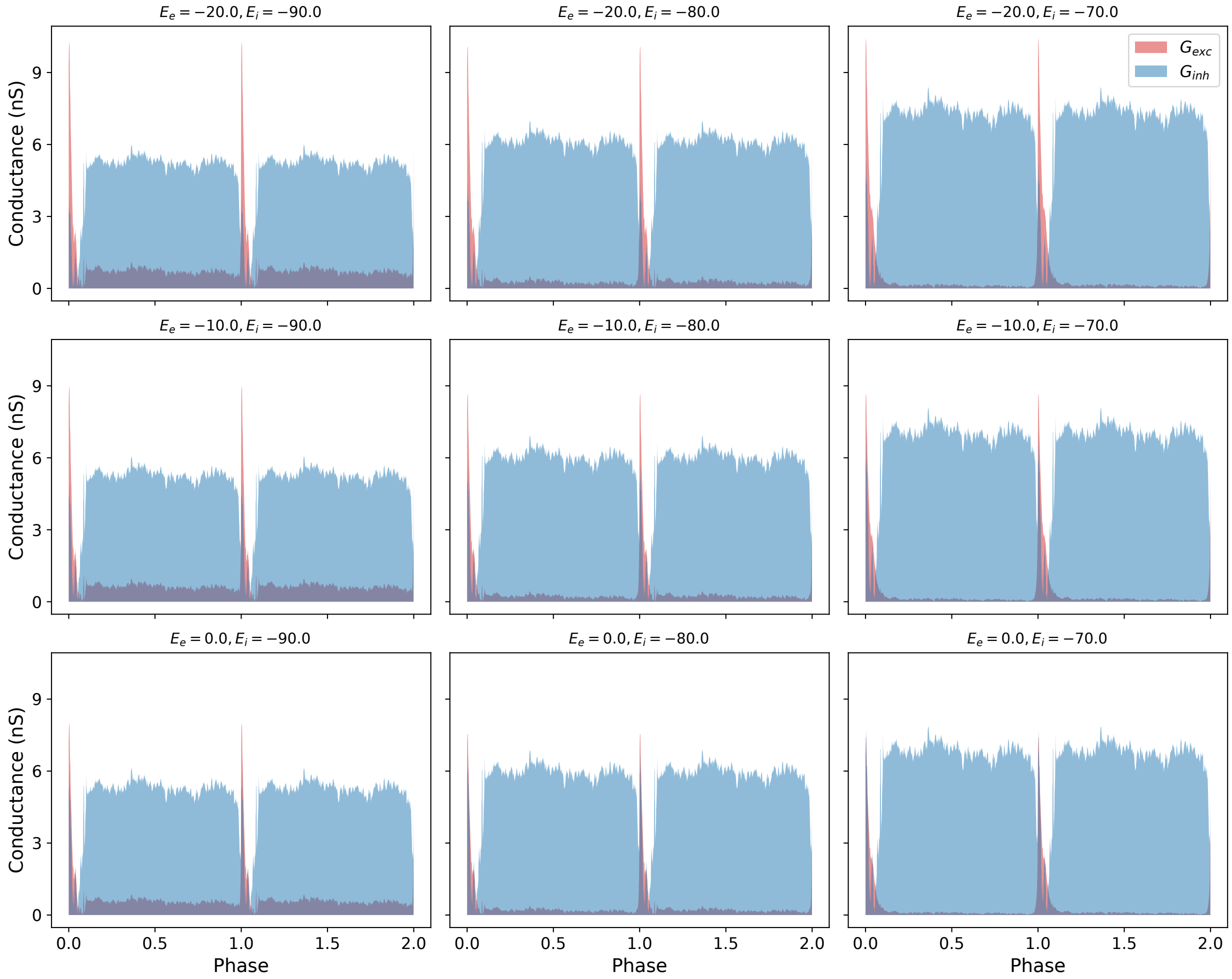

Supplemental Figure 1. Sensitivity of reconstructed conductances to variations in reversal potentials (E_e and E_i). Reconstructed excitatory (G_{exc}, red) and inhibitory (G_{inh}, blue) conductances for a representative VgluT2 inspiratory neuron were recalculated over two normalized respiratory cycles while varying the assumed excitatory and inhibitory reversal potentials. Rows show E_e values of -20, -10, and 0 mV; columns show E_i values of -90, -80, and -70 mV, spanning ±10 mV around the default values used for this sensitivity test. Automatic estimation of E_i was disabled for this diagnostic analysis so that each panel reflects the specified reversal-potential pair. The parameter sweep primarily rescales conductance amplitudes while preserving the temporal organization of the inferred synaptic inputs, including phasic inspiratory excitation and tonic expiratory inhibition.

The amplitude of the reconstructed inhibitory conductance (G_{inh}) scales with the assumed value of E_i. As E_i is shifted from -90.0 mV to -70.0 mV (moving left to right across the grid), the absolute magnitude of the

bioRxiv preprint doi: https://doi.org/10.64898/2026.05.23.727419; this version posted May 24, 2026. The copyright holder for this preprint (which was not certified by peer review) is the author/funder, who has granted bioRxiv a license to display the preprint in perpetuity. It is made available under a CC-BY 4.0 International license.

calculated G_{inh} increases. This scaling occurs as a function of the reduced driving force $(V_m - E_i)$ required to produce the observed current-voltage relationships. In all tested configurations, the temporal structure of the inhibition remains identical, showing a high-amplitude tonic component throughout the expiratory phase and a slight dip at the onset of the inspiratory burst.

Variations in the excitatory reversal potential (E_e) result in proportional scaling of the excitatory peaks. Moving from the top row (E_e = -20.0 mV) to the bottom row (E_e = 0.0 mV), the peak amplitude of G_{exc} during the inspiratory phase decreases. The timing of the excitatory burst relative to the respiratory cycle phase is preserved across all combinations of reversal potentials. The "synaptic fingerprint" of the neuron, characterized by the absence of significant excitation during the expiratory interval and the presence of a sharp inspiratory excitatory spike, is maintained in every panel of the sensitivity analysis.

The relationship between the conductances across the respiratory cycle is consistent regardless of the specific parameters chosen. The tonic nature of the expiratory inhibition persists across the entire tested parameter space. Furthermore, the relative dominance of the inhibitory conductance during the inter-burst interval compared to the excitatory conductance is a constant feature across all nine panels. The temporal organization of the synaptic inputs, including the phase-locking of the excitatory peaks and the duration of the expiratory inhibition, shows no variation across the grid of reversal potentials.

Linearity of I-V Regressions and Cross-Mode Consistency

The validity of the conductance reconstruction relies on the linearity of the relationship between membrane current and potential over the analyzed range. Supplemental Figure 2 displays the current-voltage (I-V) relationships for all representative cell types and recording modes discussed in the primary text. Data points from two distinct phases—peak inspiration ($\phi \approx 0$, red) and mid-expiration ($\phi \approx 0.5$, blue)—are fitted with linear regressions to estimate the total synaptic conductance and the current intercept. Across all neurons, including VgluT2 and VGAT populations, the observed current values follow a linear relationship with the membrane potential, supporting the steady-state assumption of the current balance equation used for the inference.

The analysis of these I-V plots reveals high consistency between current-clamp (left panels) and voltage-clamp (right panels) recording configurations. In both modes, the slopes of the regressions undergo phase-dependent shifts that correspond to the dynamic changes in total membrane conductance. For inspiratory neurons, the separation between the red and blue regression lines illustrates the recruitment of phasic excitatory drive and the modulation of inhibitory conductance. For expiratory neurons, the steepness of the slopes during inspiration (red) reflects the strong phasic inhibition received by these cells. The high degree of linearity observed in the scatter points for both recording modes indicates that the inferred synaptic conductances are not significantly distorted by non-linear intrinsic currents within the sampled voltage ranges.

bioRxiv preprint doi: https://doi.org/10.64898/2026.05.23.727419; this version posted May 24, 2026. The copyright holder for this preprint (which was not certified by peer review) is the author/funder, who has granted bioRxiv a license to display the preprint in perpetuity. It is made available under a CC-BY 4.0 International license.

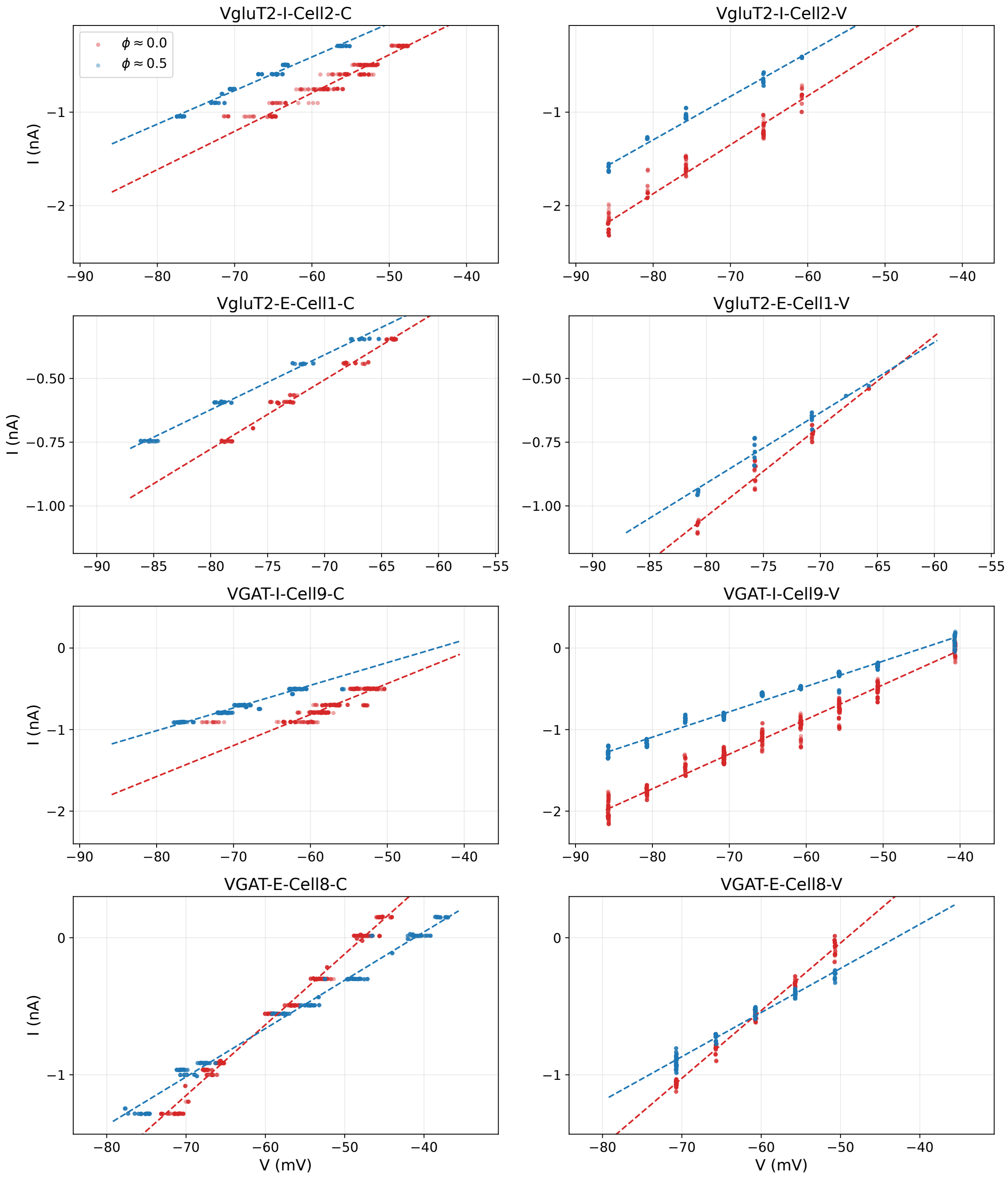

Supplemental Figure 2. Linearity of phase-binned current–voltage relationships across populations and recording modes. Each row shows I–V data for a representative neuron of one of the four populations (top to bottom: VgluT2-I, VgluT2-E, VGAT-I, VGAT-E; cell identifier indicated above each panel). Left column: current-clamp recording. Right column: voltage-clamp recording from the same neuron. Scatter points are individual current–voltage samples drawn from two narrow phase bins of the network respiratory cycle—peak inspiration ($\phi \approx 0$, red) and mid-expiration ($\phi \approx 0.5$, blue)—and dashed lines are the corresponding robust linear fits used to estimate the total membrane conductance and current intercept (cf. Figure 2). Within the sampled voltage range, the data are approximately linear in all panels, supporting the steady-state linear current balance assumption used in the conductance decomposition. Phase-dependent shifts in slope and intercept between the red and blue regressions reflect the recruitment of phasic excitatory and inhibitory conductances over the cycle.

bioRxiv preprint doi: https://doi.org/10.64898/2026.05.23.727419; this version posted May 24, 2026. The copyright holder for this preprint (which was not certified by peer review) is the author/funder, who has granted bioRxiv a license to display the preprint in perpetuity. It is made available under a CC-BY 4.0 International license.

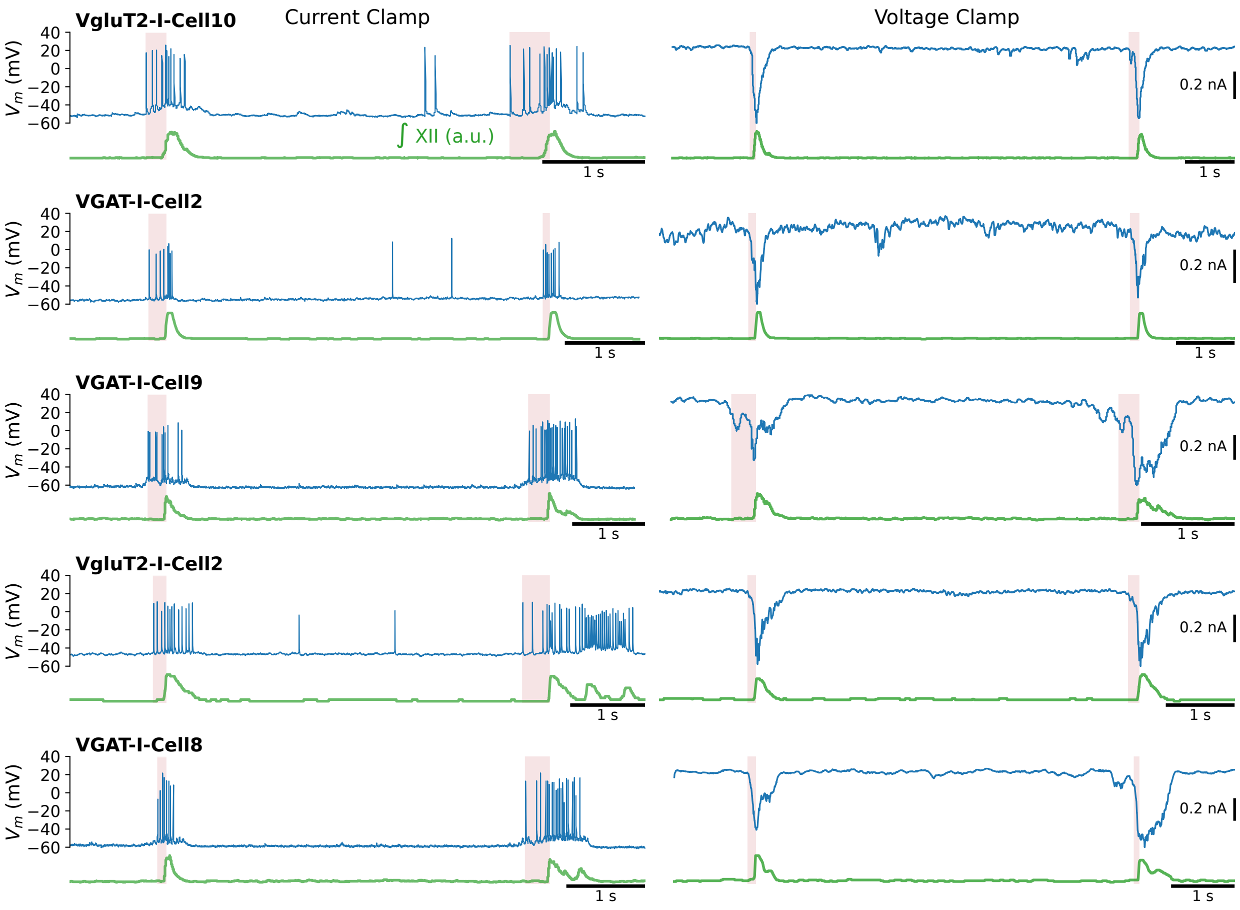

Supplemental Figure 3. Pre-Inspiratory Activity. High-resolution comparison of pre-inspiratory action potential firing and underlying synaptic drive. Left column: Current-clamp episodes show pre-inspiratory spiking (blue) preceding each network burst in the reference signal (green). Right column: Corresponding median-filtered voltage-clamp holding-current traces from the same cells show the pre-inspiratory inward current (blue) preceding the reference bursts (green). Each panel contains two adjacent reference bursts; pink shading marks the pre-inspiratory lead interval for each burst. Horizontal scale bars indicate 1 s, and Voltage Clamp panels include vertical current scale bars in native units.

bioRxiv preprint doi: https://doi.org/10.64898/2026.05.23.727419; this version posted May 24, 2026. The copyright holder for this preprint (which was not certified by peer review) is the author/funder, who has granted bioRxiv a license to display the preprint in perpetuity. It is made available under a CC-BY 4.0 International license.

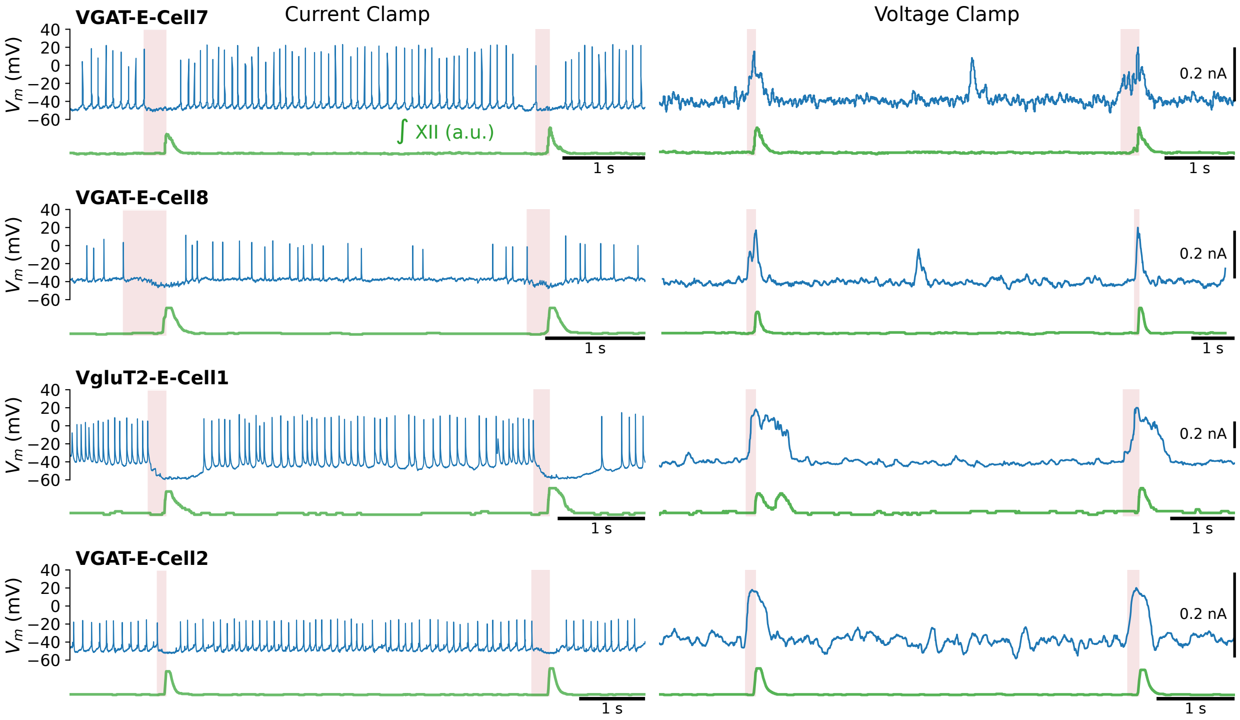

Supplemental Figure 4. Pre-Inspiratory Inhibition of Expiratory Cells. Examples of expiratory cells that stop firing before inspiratory onset and matched voltage-clamp recordings from the same cells showing a pre-inspiratory outward current. Left column: Current-clamp episodes show expiratory spiking (blue) terminating before each inspiratory burst in the reference signal (green). Right column: Corresponding median-filtered voltage-clamp holding-current traces show an outward synaptic current (blue) preceding the same inspiratory reference bursts (green). Each panel contains two adjacent reference bursts; pink shading marks the pre-inspiratory silent interval in current clamp and the pre-inspiratory outward-current interval in voltage clamp. Horizontal scale bars indicate 1 s, and voltage-clamp panels include vertical current scale bars in native units.

Pre-Inspiratory Dynamics and Phase Transitions

These supplemental panels provide representative examples of the pre-I dynamics discussed in the main text.

Pink intervals in Supplemental Figures 3–4 mark the interval between a cell-specific pre-I event and the onset of the next population inspiratory burst. The right edge of each interval is the onset of the next network inspiratory burst, detected from the smoothed integrated XII reference and aligned to its sharp rising phase. In Supplemental Figure 3, the left edge marks the beginning of pre-I spiking in current clamp or the first sustained inward deflection in the filtered voltage-clamp holding current. In Supplemental Figure 4, the left edge marks the end of expiratory spiking in current clamp or the first sustained outward deflection in the filtered voltage-clamp holding current.
